# Effect of Graphene Oxide Nanomaterials on the Durability of Concrete: A Review on Mechanisms, Provisions, Challenges, and Future Prospects

**DOI:** 10.3390/ma17102411

**Published:** 2024-05-17

**Authors:** Danula Udumulla, Thusitha Ginigaddara, Thushara Jayasinghe, Priyan Mendis, Shanaka Baduge

**Affiliations:** Department of Infrastructure Engineering, The University of Melbourne, Parkville, VIC 3010, Australia; ddudumulla@student.unimelb.edu.au (D.U.); tginigaddara@student.unimelb.edu.au (T.G.); thushara.jayasinghe@unimelb.edu.au (T.J.); pamendis@unimelb.edu.au (P.M.)

**Keywords:** durability, graphene oxide, concrete, nanomaterials, graphene, chemical attack, chloride attack, sulphate attack

## Abstract

This review focuses on recent advances in concrete durability using graphene oxide (GO) as a nanomaterial additive, with a goal to fill the gap between concrete technology, chemical interactions, and concrete durability, whilst providing insights for the adaptation of GO as an additive in concrete construction. An overview of concrete durability applications, key durability failure mechanisms of concrete, transportation mechanisms, chemical reactions involved in compromising durability, and the chemical alterations within a concrete system are discussed to understand how they impact the overall durability of concrete. The existing literature on the durability and chemical resistance of GO-reinforced concrete and mortar was reviewed and summarized. The impacts of nano-additives on the durability of concrete and its mechanisms are thoroughly discussed, particularly focusing on GO as the primary nanomaterial and its impact on durability. Finally, research gaps, future recommendations, and challenges related to the durability of mass-scale GO applications are presented.

## 1. Introduction

The premature deterioration of concrete structures creates economic, social, and environmental implications, with massive investments required for maintenance and repair when their durability is compromised [1,2,3,4,5,6]. For a concrete structure to be sustainable, it should emerge as a solution with minimal pollution and costs, whilst increasing comfort and safety throughout its designated service life [7]. Furthermore, sustainable concrete structures also use economically feasible green materials that require lower maintenance and have a higher durability [8,9,10]. Hence, sustainability in concrete construction can be successfully achieved by producing durable concrete structures with extended service life [11,12,13,14]. It is vital for concrete structures to triumph in their design functions throughout their stipulated projected service life, since the cost of concrete repairs/maintenance is significantly high, costing billions of dollars annually and adding more CO_2_ emissions during rectification construction [4,15,16].

Ordinary Portland Cement (OPC) is the most widely used cement binder in modern concrete applications. The manufacturing of OPC clinker results in utilizing massive quantities of natural resources and energy, which has an adverse impact on the environment [17,18,19,20,21]. It is estimated that 0.83 kg of CO_2_ is produced during the production of 1 kg of cement clinker, which corresponds to the cement manufacturing industry bearing responsibility for nearly 6-8% of global CO_2_ emissions [17,22,23,24]. Enhancing the longevity and efficacy of concrete structures unquestionably contributes to the global objectives of reducing CO_2_ emissions. This contribution is, in part, realized through the mitigation of repair and maintenance efforts, as well as the reduction in cement consumption in concrete, thereby promoting sustainability. However, the successful implementation of such endeavours is contingent upon the identification and adoption of economically viable, enduring solutions and techniques [24,25]. 

Numerous studies have proven that the incorporation of nanomaterials into concrete significantly improves the performance of concrete, thus resulting in enhanced durability [26,27,28]. Two-dimensional (2D) nanomaterials have garnered substantial attention in recent years, owing to their distinctive and enhanced physical properties. A defining characteristic of these 2D materials is their existence in a single atomic layer or plane, which arises from the presence of robust covalent bonds between the constituent atoms within that plane [29]. These strong in-plane covalent bonds serve as a fundamental basis for the exceptional mechanical, electrical, and thermal properties exhibited by 2D materials. Among the plethora of 2D materials, graphene stands out as one of the most prominent and extensively studied materials. Graphene is a crystalline allotrope of carbon, comprising a monolayer of carbon atoms arranged in a hexagonal lattice. GO is obtained through the oxidative exfoliation of graphite, a layered carbon allotrope [30,31,32]. During this process, various oxygen-containing functional groups are introduced onto the graphene lattice, altering its chemical composition and reactivity. This modification yields a material distinct from pristine graphene, with notable characteristics such as improved solubility in aqueous solutions [32]. The diverse chemical properties of graphene oxide have kindled considerable interest in its utilization across various fields. 

GO and graphene-based nanomaterials have also acquired substantial interest due to their superior durability enhancements in cementitious composites and other material science applications [23,31,33,34]. However, whilst GO has gathered considerable attention in the field of material science, research specifically focused on its application in GO-reinforced concrete remains relatively limited compared to other nanomaterial composites. Although GO has exhibited exceptional properties such as high tensile strength, impressive thermal and electrical conductivity, and excellent mechanical properties, its integration into concrete matrices for reinforcement purposes is an area that requires further exploration. The potential benefits of utilizing GO as a reinforcing agent in concrete are immense, including enhanced durability, improved mechanical strength, and increased resistance to cracking. However, due to several factors, including the complexity of the manufacturing process, the dispersibility issues of GO, and the lack of consistent results, research in this specific area is relatively scarce. The majority of the studies have primarily focused on incorporating GO into cementitious composites, such as mortars or pastes, where it can act as a cement replacement or supplementary material, thereby enhancing the overall performance of the composite. GO-reinforced concrete holds great potential for revolutionizing the construction industry; further investigations and dedicated research efforts are necessary to fully exploit its benefits. By addressing the current knowledge gaps and expanding research initiatives, the untapped potential of GO in reinforced concrete can be explored and validated, leading to the development of high-performance and sustainable construction materials.

Considering the higher-performance enhancements observed by incorporating nanomaterials into concrete, this study explores the role of nanomaterials, with a primary focus on the durability enhancements of incorporating graphene oxide into concrete. This review aims to provide a holistic understanding of the prevailing durability applications, the key durability failure mechanisms of concrete, the transportation mechanisms, the chemical reactions involved in weakening the durability of concrete, and the significant durability improvements in GO-reinforced concrete and insights into the challenges and opportunities in commercializing GO for durable concrete applications. A thorough literature review is carried out using experimental work on the durability of GO and graphene-based nanomaterial-incorporated concrete, the durability enhancement from advanced nanomaterials is extensively investigated, and the primary durability-deteriorating mechanisms of concrete structures, including various forms of chemical attacks and their Australian Standard code provisions, are comprehensively reviewed. 

## 2. Durability

The durability of concrete can be defined as the ability to resist adverse service life situations such as acid attacks, biological attacks, chlorine attacks, carbonation, sulphate attacks, alkali–aggregate (silica) reaction, etc., and physical weathering phenomena during its service life [35,36]. The deterioration of concrete is significantly severe when these adverse factors combine, compared to the action of a single influence [37,38]. Despite a substantial amount of studies undertaken on concrete durability over the years, asset owners demand to further enhance the durability of concrete structures since it significantly affects the service life and maintenance cost [2,18]. Durable concrete structures would lead to a prolonged service life, which results in a reduction in costs and maintenance and a significant amount of CO_2_ emission savings [37,39]. Concrete is vulnerable to a wide range of deterioration mechanisms, both physically and chemically, but chemical deterioration leads to an accelerated reduction in service life and is considered the most challenging. The chemical deterioration of concrete can occur due to various factors and the ingress of harmful agents such as sulphate ions (SO_4_^2−^), carbon dioxide (CO_2_), and chloride ions (Cl^−^), which are considered the most significant and common deterioration agents of concrete [40,41]. Additionally, there are other factors that can lead to the deterioration of concrete, including calcium leaching, alkali–silica reaction (ASR), freeze-and-thaw cycles, and bacterial attacks [3,5,16,42,43,44]. The engineering properties of concrete durability are mainly influenced by the permeability and diffusion medium of the concrete [18,45,46,47]. Dense concrete structures have fewer voids and pores whilst favouring a higher load-carrying capacity [48]. Concrete with fewer voids obstructs the permeation of harmful liquid chemicals and water, thus resulting in a longer service life [18,21,37,46]. 

Researchers have incorporated nanomaterials into concrete and investigated the durability of concrete and its microstructural changes. Samchenko et al. [38] optimized the composition of cement paste using combined additives of Alumoferrites and gypsum, which led to a dense microstructure. An increase in the corrosion resistance of the hardened cement paste was observed owing to its dense microstructure [39]. M. Zhang et al. [44] experimented with the combined effect of Cl^−^ erosion and carbonation on concrete doped with nano-SiO_2_ and nano-ZnO (1–2% BWOC). Nano-silica showed better resistance to the combined effect of Cl^−^ erosion and carbonation over nano-ZnO, whilst the microscopic tests revealed that nano-SiO_2_ was denser than nano-ZnO, implying a relationship between pore structure and durability. Kreem et al. [46] studied the effect of curing methods on concrete with various contents of OPC and w/c, investigating absorption, density, and porosity in relation to the durability of the concrete. The findings revealed that the durability of concrete is more related to w/c, where the reduction in water content reduces the volume of capillary pores, thus reducing water absorption as well as porosity, creating denser structures [46]. The Hornibrook bridge, Australia, is an excellent field example for reinforced concrete piles designed to withstand a long service life, despite being located in a zone with very high chloride exposure [4]. Visual observations and random tests conducted on several concrete samples highlighted the physical integrity, low permeability, high pH (around 12), and lack of voids in the concrete [4]. One of the key effective strategies adopted by engineers is to address durability complications during the design and construction phase, thus capitalizing on significant savings on maintenance cost, time, and CO_2_ over the life span of the structure. Addressing the aggressive agents and transport mechanisms involved in concrete structural deterioration is of utmost importance in structural design to guarantee a healthy service life for structures [5,15,18,49]. 

### 2.1. Transport Mechanisms in Concrete

The transportation of liquids, gases, and ionic species through concrete is as a primary factor contributing to durability challenges as it possesses the potential to significantly impact the structural integrity of concrete structures [5,21]. This is due to the interactions that occur between these infiltrating substances and the constituents of concrete as well as the pore water within the concrete matrix. The movement of these elements within the concrete usually occurs via cracks and concrete pores, generally due to various combinations of humidity, pressure (air/water), concentration, or temperature differentials of solutions. Restricting the transportation mechanisms within the concrete enhances concrete durability significantly [16,50]. The cardinal transport methods by which aggressive agents ingress into the concrete causing deterioration include absorption, diffusion, capillary action, and permeation [5,47]. Diffusion allows the mass flow due to the differences in concentration gradients on two given points to neutralize the effect of the concentration gradient [22,51,52]. Diffusion is progressed much faster in gases, followed by liquids, whilst diffusion is slowest in solids. In concrete systems, chemical reactions that take place between penetrating matter and concrete affect the diffusion process, which is a key factor to consider in concrete durability [52]. Absorption refers to the transportation of liquids within porous solids that takes place due to the surface tension present in the capillaries. Absorption signifies the bulk uptake of water, which is accountable for the rise in moisture content in concrete. Absorption directly correlates with the pore structure and moisture condition of porous solid concrete. A higher-refined pore structure results in a reduction in the water absorption of concrete, thus enhancing its durability [53]. Sorptivity refers to the ingress of water into concrete through capillary rise from the pores of the concrete in contact with the liquid phases [51,54]. However, since the actions of both absorption and sorption happen simultaneously, a combined effect known as the capillary effect is considered to quantify absorption in dry concrete [53,55]. Temperature gradients, also known as the Soret effect, and chemical activity effects within the system might also affect the motion of charged ions, especially in saturated conditions [54].

### 2.2. Chloride Ingress

Despite concrete being the most widely used construction material for a wide range of structural applications, it is brittle in nature and signifies poor performance against tensile loads [23,56]. Steel rebars are routinely used as reinforcement to mitigate unfavourable tensile performances and have proven to be an effective and economical reinforcement in concrete structures for many years. Furthermore, using steel reinforcement improves the overall strength of the concrete structures whilst delaying crack development in concrete [51,57]. Unsatisfactory concrete cover quality, poor design practice, presence of aggressive agents and structures in coastal environments exposed to seawater, high temperature, and moisture encourage chloride ingress into the concrete, thus initiating corrosion [37]. Chloride ions can permeate into cementitious concrete exposed to chloride salt solutions through diffusion, wicking, and absorption processes [22,42,51,58]. 

Two major problems arise with reinforcement corrosion in concrete structures [59]:

1. Loss of the cross-section of reinforcement significantly reduces the load-bearing capacity, thus impacting the safety of the structure.
2. Cracking and spalling accelerate damage propagation with respect to the concrete cover, which may pose a direct safety threat to passing traffic nearby. The volume expansion that takes place due to corrosion is usually 1.5 to 6.5 times that of iron.

The corrosion of steel reinforcements in concrete structures predominantly occurs owing to two primary mechanisms: chloride-induced corrosion and carbonation-induced corrosion. Among these mechanisms, chloride-induced corrosion stands as the most prevalent and frequently observed deterioration process affecting concrete structures, due to the high volume of concrete structures present in coastal areas [43,60]. Chloride ingress into concrete structures causes extensive damage, which may eventually lead to failure, resulting in a reduced service life [2,51,61]. The annual cost of rectifying structures due to corrosion worldwide is over USD 1.8 trillion. In Australia, the repair or replacement associated with steel corrosion costs the economy an estimated AUD 13 billion per year. In the future, corrosion damage will be problematic to not only coastal environments but for various inland structures as well due to increased CO_2_ concentrations, temperatures, and humidity as a result of global warming and climate change [49]. Coastal environments contain aggressive soil conditions that accelerate the corrosion damage to structures. Moreover, concrete structures exposed to tidal or splash conditions in seawater amplify their sorptivity, thus accelerating corrosion initiation, whilst climate change can result in the risk of corrosion increasing in regional areas as well [16,62].

**Figure 1:**
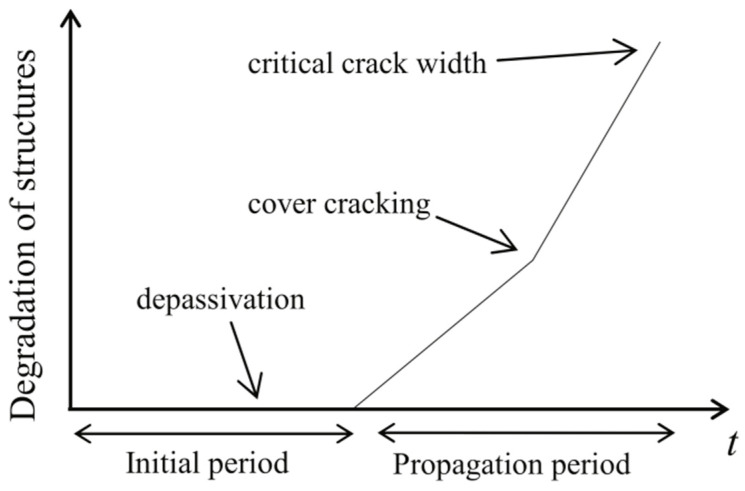
Illustration of corrosion phases in steel reinforcements [64].

The corrosion of steel reinforcements takes place in two phases: namely, the corrosion initiation phase and the corrosion propagation phase [63]. The corrosion initiation phase represents a critical stage wherein the alkalinity of the concrete gradually diminishes to a level that triggers the commencement of active corrosion through the depassivation of steel rebars. The corrosion propagation phase relates to the period from corrosion initiation to a point where corrosion products cause a failure in the surrounding concrete, such as cracking or spalling, as shown in Figure 1 [49,57]. 

Once corrosion initiates, very limited measures can be adopted to rectify the issue, with the exception of methods such as cathodic protection [58]. However, it is indeed possible to build concrete structures in which steel reinforcement will not easily corrode, but it comes with a massive cost premium. The depth of concrete cover specified by Australian standards AS3600 in certain harsh environments is not economically viable with the cost premium involved, whilst, in most construction projects, it is difficult to justify the additional cost of durability enhancements [65]. The Gateway Bridge in Brisbane, Australia, is one such example where stainless steel reinforcing bars were utilized with highly compacted and cured high-strength concrete, which ensured a design life of 300 years despite the high costs [66].

#### 2.2.1. Corrosion Mechanisms

Initially, steel reinforcements in concrete are protected by a very stable oxyhydroxide film (owing to the high alkalinity of concrete pore liquids), also known as the passive layer which prevents the free chloride ions available in the system from interacting with the steel reinforcement, thus inhibiting corrosion [59]. The thermodynamic data of iron (Fe) imply that the insoluble oxides that construct the passive protective film are thermodynamically stable in the alkaline environments of concrete (pH 12,13) [57]. Primarily, there are two main corrosion mechanisms identified that lead to the contamination of the passive layer, initiating corrosion in the presence of oxygen and humidity [59,65].

1. Uniform Corrosion (Carbonation-induced): Carbonation leads to a reduction in the pH of the concrete. Carbonated concrete in contact with the reinforcement surface contaminates the passive layer, initiating corrosion in the presence of humidity and oxygen. In this occurrence, the steel corrodes uniformly [57].
2. Localized Corrosion (Chloride-induced): Free chloride ions, when present in sufficient quantities, can break down the passive oxide layer on steel. Localized corrosion can develop when an adequate concentration of chloride ions becomes sequestered in the vicinity of steel reinforcements, despite upholding the alkaline properties of the concrete [59].

#### 2.2.2. Chloride-Binding Mechanisms

To further investigate chloride’s interaction with respect to the corrosion of steel rebars, it is fundamental to understand the influence of bound chlorides and free chlorides in a cementitious system. When chlorides penetrate the concrete cover externally, they tend to be adsorbed into the main cement hydration product, calcium silicate hydrate (CSH), owing to their positive surface charge. The surface charge of CSH is primarily governed by the segregation of cementitious phases, the adsorption of ions, and the charge associated with the significantly high Ca^2+^ ions present in the pore solution [67,68]. When chloride ions penetrate the concrete, a percentage of them are bound, whilst the remaining free chloride ions passage through the pore solution with the aid of different transport mechanisms. Chloride binding is the result of chloride ions’ physical/chemical interactions with cement hydration products. However, once a certain threshold of free chloride ion concentration is exceeded, the depassivation of the steel rebar occurs, which sanctions the chloride ions to react with the elements present in steel, mainly Fe^2+^ ions. The precise value of this threshold is still subject to ongoing research, whilst most researchers agree that the threshold depends on the free chloride/total chloride content or the ratio of the free chloride to the hydroxyl ion content (Cl^−^ = OH^−^) [58]. 

The accurate determination of the free chloride concentration is imperative in estimating various concrete service life parameters such as the corrosion initiation period, the remaining service life, etc. [37,43]. Once chloride reacts with steel, it forms Fe(OH)^2^ deposits along the steel reinforcement, which leads to an expansion of the concrete, whilst the oxidation which occurs within the cell leads to a significant reduction in the cross-section of the steel rebar [65]. This action will induce microcracks, which will further accelerate chloride-induced corrosion whilst exponentially increasing the rate of premature deterioration of concrete structures. Moreover, cracks in concrete create preferential pathways for aggressive corrosion agents such as Cl, H_2_O, SO_4_, and CO_2_ to further deteriorate the concrete, thus reducing the service life of structures. Hypothetically, provided that no cracks are present, corrosion initiation and propagation in concrete are usually a function of the permeability, cover thickness, and resistivity of the concrete cover [2,3]. 

Furthermore, a fraction of the different aluminate phases present in cement, namely, C_3_A and C_4_AF, react with chloride ions to form Friedel’s salt (FS: Ca_4_Al_2_(OH)_12_Cl_2_·4H_2_O), Kuzel’s salt (KS: Ca_4_Al_2_(OH)_12_Cl(SO_4_)_0.5_·5H_2_O) and oxychloride phases [37,43,69,70]. However, only a highly chloride-concentrated environment can give rise to Kuzel’s salts and oxychloride phases, whilst the formation of Friedel’s salt lies within the critical chloride concentration in concrete [43,69]. Moreover, C_3_A content and exposure temperatures also have a very strong impact on the chloride threshold [58].

The remaining chloride ions present in the pore solution are free chloride ions, which tend to diffuse through the concrete and depassivate the steel rebar, initiating corrosion [43,69,70]. Hence, one of the strategies to eliminate or prolong chloride-triggered corrosion is to optimize the chloride-binding ability of concrete, which, in return, would lessen the number of free chlorides present in the pore solution. Moreover, the reaction between chloride ions with tricalcium aluminate (C_3_A) and mono sulphates (Afm) forms Friedel salts, which also highlights the fact that cementitious materials with a rich alumina content encourage the chloride-binding ability of cement. Furthermore, the concentration of Ca^2+^ in the cement matrix is equally important for the formation of calcium chloroaluminate, which leads to the formation of Friedel’s salt. 

Given the high amorphous silica content present in many traditional supplementary cementitious additives, it is unfavourable for the formation of Friedel salts due to its overconsumption of Ca^2+^ ions upon hydration. Once the maximum chloride-binding capacity is reached, a dynamic equilibrium is maintained between the free chlorides and the bound chlorides. Numerous studies have identified that this equilibrium can be altered by changes to the pore solution’s chemistry, such as controlling the concentration and the pH [45,70].

### 2.3. Sulphate Attack

Agricultural locations, coastal/marine regions, wastewater treatment plants, and tunnels are a few of the common scenarios that suffer severe sulphate-induced concrete degradation [54,69,71,72]. Primarily, there are three common modes of sulphate deterioration in concrete.

**Figure 2:**
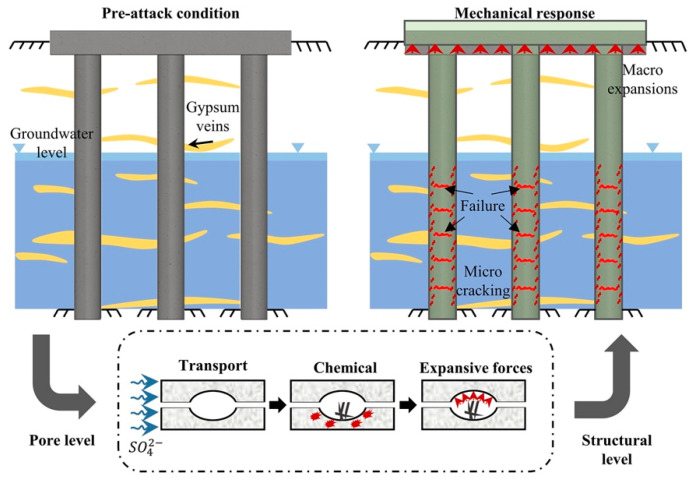
Schematic representation of the processes involved in sulphate attacks [54].

1. The first mode, also known as the acidic type of sulphate attacks, indicates the consumption of hydrated cement paste, which weakens the cohesion between the aggregates and the cement paste. This causes the erosion of the granular mass in the cement paste, thus exposing the aggregates. As illustrated in Figure 2, the main mode of failure is owed to the formation of gypsum, which directly corresponds to a loss in mechanical performance and the cross-sectional area of the exposed structure [73,74].
2. The second mode of deterioration, also known as the expansive type of sulphate attacks, relates to the expansion and cracking of the concrete that take place when sufficient quantities of hydrated aluminate phases react with the sulphate ions in high-pH media, forming tricalcium sulpha aluminate hydrate (ettringite) [71,73,74].
  An expansion-type sulphate attack results in the spalling and cracking of concrete, layer by layer, resulting in a loss of the cross-sectional area and mechanical performance of the exposed structure.
3. The third mode of deterioration, also known as the peeling type of sulphate attacks, relates to the shelling of the concrete surface in impending layers.

In its initial stages, a sulphate attack on concrete will result in the hardening of the cement matrix due to the initial filling of voids and pores by crystals of gypsum or ettringite, which increase the overall density of the cement or mortar [54]. These newly formed crystals cause expansion directly by anisotropic growth or indirectly by increasing the pore water pressure, giving rise to internal stresses which ultimately collapse the concrete. Ettringite occupies a higher-volume expansion than gypsum (the volume of the solid is more than doubled), which leads to microcracks inside the cement matrix due to its inability to handle the expansive stress [75]. Furthermore, upon the continuation of this behaviour, CSH decomposes due to decalcification, which reduces the mechanical performance of the concrete [71,73]. Furthermore, when a sulphate attack is coupled with drying–wetting cycles, due to the frequent disturbance of the state of equilibrium within the cementitious matrix (i.e., drying and wetting actions), an accelerated impact on the sulphate deterioration mechanisms can be observed [37,72]. 

Acidic rainwater materializes due to the dissolution of SO_2_, CO_2,_ and NO_x_ in rainwater, which results in the formation of acids: namely, carbonic, sulfuric, and nitric acids. When concrete overlays are exposed to acidic rainwater, the pH. of the water is lowered towards the acidic range (pH ≈ 4–5), thus compromising the cement paste by supporting dissolution and neutralization [76]. Furthermore, in coastal/marine regions where the groundwater table is mostly shallow, concrete structures are often prone to salt scaling. Concrete structures constructed in marine coastal areas directly expose their foundations to the high concentration of sulphate and chloride ions present in groundwater [42]. These harsh ions can enter the erected structure via its foundation or be sucked up due to the wicking phenomenon. When the contaminated water evaporates, it leaves the crystalized salts on the concrete cover [77]. Repeated cycles of this action together with the influence of environmental conditions such as heat and humidity accelerate the formation of these destructive salts, resulting in the consequent crumbling, scaling, or salt blistering, which damages the surface of the concrete, exposing the aggregates. This action is amplified within the tidal/splash zone, in which marine concrete structures are even more vulnerable to deterioration due to the impact of wet and dry cyclic actions, resulting in rapid dissolution and deposition of these salt crystals [78]. Sulphate-resisting ASTM C 150 Type V cement, despite having some resistance to sulphate degradation, is still vulnerable to sulphate attacks [79]. However, there are several factors that have a direct relationship to the rate and intensity of sulphate attacks [54,80,81].

1. The mineralogical phases of OPC—namely, C_3_A, the C_3_S/C_2_S ratio, and C_4_AF –directly affect the rate of sulphate ingress into the concrete.
2. The sulphate type and concentration also contribute to the accelerated deterioration of concrete. Another important element that has a noteworthy impact on the magnitude of concrete deterioration is the cation associated with the sulphate ion.
3. The quality of concrete is the most important factor that enables sulphate resistance whilst promoting the durability of the concrete.

Hence, concrete design and construction based on reducing the permeability and transport of these hazardous ions tend to inhibit the intensity of sulphate attacks [37]. Furthermore, since the harsh ions enter the concrete via its cover, added layers of concrete cover protection such as grout, epoxy, and polyurethanes are found to be effective in suppressing a sulphate attack [82]. Since a sulphate attack can only take place in an aqueous medium, the area of the concrete foundation most vulnerable to the sulphate attack is the area within or just above the groundwater table. Progressive research related to the impact of sulphate ions on the integrity of concrete is still in progress, primarily due to the rapid modifications witnessed in the cement manufacturing industry. 

### 2.4. Carbonation

The CO_2_ present in the atmosphere diffuses into concrete to react with calcium-bearing phases in the presence of water to form CaCO_3_ (calcium carbonate). This reaction reduces the pH of the pore solution to below 10 due to its acidic nature, which depassivates the steel reinforcement, initiating corrosion [16]. The carbonation of concrete indicates a chemical reaction between the calcium ions (Ca^2+^) present in the hydrated cement products and carbonic acid (H_2_CO_3_) to form CaCO_3_. Ca^2+^ ions are prevalent in cement products such as CSH, Ca(OH)_2_, etc., whilst carbonic acid is a result of the CO_2_ in the atmosphere reacting with water [6,16]. The transport mechanisms primarily involved in carbonation include diffusion (gas permeability) and absorption (water) [2]. The carbonation of concrete is dependent on the availability of CO_2_ and water, whilst its speed depends on the rate of its reaction with the cement-hydrated products. The microstructure and connectivity of pores are identified as the key paths through which CO_2_ diffuses into concrete [6]. Carbonation escalates the shrinkage of concrete, which results in the formation of cracks, thereby accelerating concrete deterioration [37]. The chemical reactions involved in the formation of CaCO_3_ are depicted below.

CO_2_(gas) + H_2_O = H_2_CO_3_(aq)

Ca(OH)_2_ + H_2_CO_3_(aq) = Ca_2_CO_3_ + H_2_O

3CaO.2SiO_2_.3H_2_O + 3H_2_ CO_3_ (aq) = 3CaCO_3_ + 2SiO_2_ + 3H_2_O

4CaO.Al_2_O_3_.13H_2_O + 3H_2_CO_3_(aq) = 4CaCO_3_ + 2Al(OH)_3_ + 10H_2_O

The products formed as a result of carbonation alter the chemical reactivity of the cement hydrate paste and the permeability and pore structure of the concrete [83]. Moreover, the binding capacity of aggressive ions is also impacted due to carbonation. Chloride ions that were initially physically adsorbed or chemically bonded are released during carbonation, resulting in an increased free chloride ion concentration in the matrix [84]. The combined action of carbonation and the contamination of the chloride equilibrium in concrete are considered the most severe forms of corrosion attacks in today’s practice [16]. The key driving force of carbonation is the CO_2_ concentration gradient, which controls the ingress of CO_2_ into the concrete pore system via diffusion. Hence, the carbonation rate can be controlled by restricting the diffusion of CO_2_ into concrete [16,21,47].

## 3. Durability Insights on Codes and Provisions

It is imperative to highlight that durability cannot be reduced to a single property; instead, it encompasses a collection of properties and provisions necessary for structures to achieve their intended service life under specific exposure conditions. While the strength of concrete is vital for design capacity, stability, and structural integrity, it is equally important to consider the various factors that impact the durability of concrete structures. The implementation of codes and provisions is essential, as they ensure that all the norms, recommendations, and standards necessary to protect public health, safety, and welfare are met in the design, construction, repair, maintenance, and rehabilitation of structures. Having a holistic understanding of the codes and provisions related to concrete durability is crucial for steering structural research and aligning it with regulatory requirements. 

Various guidelines and procedures have been proposed by different nations to preserve the durability of concrete structures against chloride attacks, carbonation, and sulphate attacks during new concrete construction [6,12,85]. AS 3600, AS 1379, and AS 5100.5 are Australian standards that play important roles in the design, production, and durability aspects of concrete structures in Australia. AS 3600 covers various aspects of concrete design, such as material selection, mix design, reinforcement, structural design principles, and construction practices. Whilst AS 3600 does not specifically focus on durability, it includes provisions related to durability considerations, such as concrete cover requirements and exposure classifications. AS 1379 covers aspects such as concrete constituents, mix design methods, testing procedures, and quality control. It sets standards for the concrete mix’s proportions, strength, workability, and other properties to ensure the desired performance and durability of the concrete. AS 5100.5 incorporates the exposure classification system for different environments, as shown in Table 1, including the exposure classifications for concrete in sulphate, acidic, and saline soils, and provides guidelines for concrete quality, curing, chemical content, reinforcement cover, abrasion resistance, cycles of freeze and thawing, and the effects of stray current corrosion [82]. It also includes additional durability requirements related to alkali–silica reactions, the protection of fixings, aggressive soil, and groundwater. AS 5100 incorporates an exposure classification system that encompasses five surface and exposure environments for different structural members. These environments include members in contact with the ground, as well as interior, above-ground exterior, water, and other environments. For above-ground exterior environments, AS 5100 distinguishes between five different climatic conditions: near-coastal, coastal, inland arid, temperate, and tropical. 

While AS 1379 and AS 3600 may prioritize mechanical strength as the primary criterion for acceptance, possibly considering other parameters such as lower shrinkage in certain cases, the same cannot be said for bridges and other major infrastructures governed by AS 5100 [15]. In AS 5100, both minimum strength and enhanced durability provisions are essential requirements [82]. This highlights the recognition that structures like bridges, due to their critical nature and exposure to various environmental conditions, require not only sufficient strength but also enhanced durability to ensure long-term performance and longevity. To achieve durable concrete structures consistent with the in-service exposure conditions, various properties and provisions are considered in AS 5100.5. These include the following:

1. Minimum cementitious material content: The standard specifies the minimum amounts of cementitious materials (such as cement, fly ash, or slag) to be used in the concrete mix.
2. Maximum (W/C) ratio: It sets a limit on the ratio of water to cementitious materials to ensure sufficient hydration and strength development while minimizing shrinkage and permeability.
3. Maximum permeability limits: The standard includes criteria such as the maximum volume of permeable voids (VPVs), which restricts the number of interconnected pores in the concrete, reducing the potential for hazardous substances to penetrate.
4. Minimum compressive strength: The standard defines the minimum strength requirement that the concrete must attain to ensure structural integrity and load-bearing capacity.
5. Minimum cover thickness: It specifies the minimum thickness of concrete cover over a reinforcement to protect it from corrosion and other potential sources of damage.
6. Types of cementitious materials: The standard addresses the use of supplementary cementitious materials (SCMs) like fly ash or slag, which can enhance the durability and long-term performance of concrete.
7. Limitations on soluble salts: The standard considers the maximum allowable content of soluble salts in the concrete mix to prevent potential deleterious effects on durability.
8. Limitations on alkali–aggregate reactivity (AAR): The standard addresses the potential for alkali–silica reactions or other forms of AAR that can lead to cracking and reduced durability.
9. Shrinkage limitations: It imposes restrictions on the amount of drying shrinkage, which helps to control cracking and maintain long-term durability.

In the context of bridges and major infrastructures, specifying a minimum cementitious material content can help mitigate the potential negative impacts arising from inadequate production and construction practices. This approach ensures that the concrete remains highly workable while achieving optimal densification of the concrete microstructure, minimum strength, and low permeability. These characteristics are essential to meet the maximum limits of the VPV and satisfy the required 100-year-long design and service life, thereby enhancing durability. Measures such as replacing cement with SCMs, controlling the w/c ratio, increasing the thickness of the concrete cover, and using concrete coatings (paints, resins, tar) or their combinations are commonly practiced in modern civil construction to address durability issues [57,86]. The utilization of supplementary cementitious materials in current concrete durability investigations is complemented in AS 5100.5, as they can significantly improve the transport and deterioration properties of concrete, thereby enhancing durability [82,87]. 

Durability provisions within different codes exhibit substantial variations, primarily due to the diverse types of environmental factors, regional conditions, and exposure conditions encountered [88]. It is essential to consult and adhere to the relevant code requirements within a specific demographic to ensure compliance with the relevant building permits and licenses. While some nations may not have their own structural design norms, they adopt the design codes of other developed countries instead. It is important to gain insights into the most effective and economical design practices when multiple codes are used.

## 4. Recent Developments in Concrete Durability Using Nanomaterials

**Figure 3:**
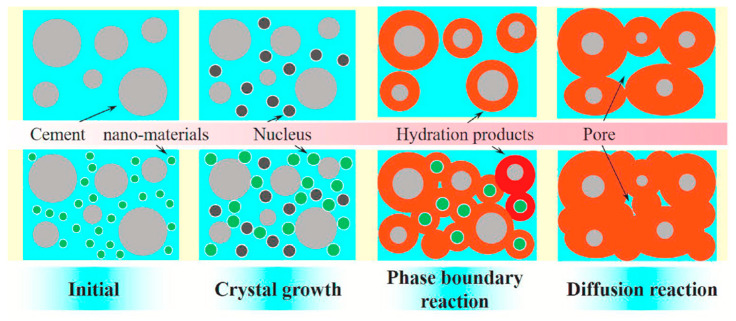
Illustration of improved nucleation effect using nanomaterials [94].

A rapid rise in research related to improving the properties of conventional concrete using nanomaterials has been seen during the past decades [86,89,90,91]. Many studies have proven that nanomaterial incorporation is a successful method of significantly improving the fundamental performance properties of concrete, whilst emerging as a catalyst enabling green building technologies [37,86]. Carbon-based nanomaterial additions to cement/concrete have proven to improve mechanical performance, microstructure, and transport properties (i.e., water/gas permeability, sorptivity, etc.) [10]. Furthermore, studies related to nanomaterials in concrete have paved the way for researchers to understand the complicated structural integrity of concrete at a nano/micro-scale. Nano-additives incorporated into concrete have significantly reduced the volume of the pore spaces, thus reducing permeability and water absorption, as depicted in Figure 3, which are promising attributes for resisting the ingress of various aggressive agents such as chlorides, sulphates, CO_2_, and water which deteriorate concrete structures [32,92,93,94]. These nanofillers anticipate a better performance by directly interacting with CSH gel that is identical in size. The influence of 1D and 2D nanofillers improves the hydration rate due to their large surface area, which improves reactivity. The mechanism of improving the mechanical and durability properties of concrete using nanomaterials is mainly attributed to the nucleation effect, the filling effect, the pozzolanic effect, the bridging effect, and ITZ enhancement [91,92,95,96].

A. Hosan et al. [97] investigated the changes in the microstructure of the ITZ with the addition of nano-calcium carbonate and nano-silica. The results indicated a significant compact microstructure with reduced porosities and fewer cracks around the ITZ area when compared with the control sample, indicating the additional formation of CSH due to pore filling and pozzolanic reactions of the nano-additives. Various studies have been conducted to understand the properties of carbon nanotubes, nano-titanium oxide, graphene oxide, nano-silica, and nano-aluminium oxide of different proportions in concrete [32,86,97,98]. The growing trend of incorporating SCMs and nanomaterials in concrete to achieve sustainable concrete solutions must be comprehensively assessed with respect to its reactivity, mechanical properties, microstructural properties, and durability [67]. One such emerging nanomaterial that highlights a massive competitive edge in all the desirable properties of conventional concrete is graphene oxide (GO).

### 4.1. Graphene Oxide

Graphene oxide emerges as an outstanding nanofiller material due to its exceptional properties as a construction material. The presence of diverse functional groups such as OH, COOH, CC, etc., alongside its large aspect ratio and atomic thickness enable GO to act as a physical filler with mechanical interlocking and bridging effects that increase coherence via authoritative covalent bonding whilst also providing a large surface area for the interaction with the cementitious matrix [31,86,99]. Graphene oxide owns the superior advantage of being hydrophilic in water, enabling the ease of dispersing into the microstructure of cementitious composites, which is a major challenge when working with other concrete nano-additives such as carbon nanotubes (CNTs) [32]. Numerous studies have demonstrated that the addition of GO with weight percentages ranging from 0.1% to 0.12% has resulted in concretes with enhanced strength and durability [86,93,100,101].

**Figure 4:**
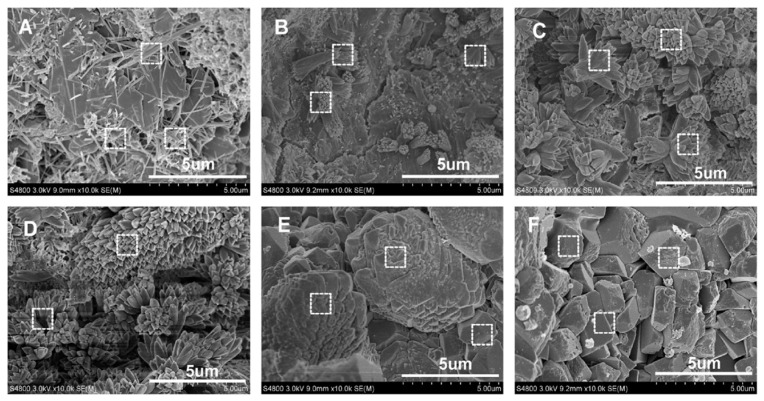
SEM images of cement composites after 28 days, mixed with various concentrations of GO: (**A**) no GO; (**B**) GO0.01%; (**C**) 0.02%; (**D**) 0.03%; (**E**) 0.04%; and (**F**) 0.05% [34].

The strength gain mechanism of GO in cementitious composites is primarily driven by its ability to generate multiple hydration nucleus sites, also described as the template effect. Accordingly, these nuclei, over a period, tend to form flower-like hydration crystals that enhance the packing density of the composite, as depicted in Figure 4 [34]. 

The addition of GO also reduces the average pore size whilst transforming large macropores into small micropores, thus enhancing the total porosity of the concrete [32,67]. Furthermore, incorporating GO tends to repair microcracks that take place within the concrete whilst regulating the aggregation of hydrated products, thus making the concrete more dense and regular [67]. Research studies conducted by incorporating graphene–cement–concrete composites have shown that the existence of graphene nanoparticles significantly changes the microstructure of cement composites, as they are directly related to the strength gain, porosity, and chemical resistance of the concrete. X. Li et al. [102] incorporated small quantities of well-dispersed graphene oxide (GO) into cement mortar, investigating the improvement in its mechanical properties, and identified that the improvements were due to pore structure refinement and ITZ microstructure densification. D. Lu et al. [103] experimented with a targeted approach to improve the microstructure of ITZ by coating GO on recycled concrete aggregate (RCA), and this resulted in a denser microstructure with fewer pores and thinner ITZ with fewer microcracks than the control sample. These findings imply that the hydrophilic functional groups of GO promote cement hydration at the ITZ. Liu C. et al. studied the mechanical and durability properties of steel fibre-reinforced concrete specimens by incorporating 0.03 wt.% GO, and, after successful curing for 28 days, they showed a 53.8% lower chloride ion penetration depth than the reference sample. This highlights the refining of the pore size and its distribution by promoting the transformation of large micropores to small micropores using GO. Table 2 summarizes a review of studies conducted incorporating GO into concrete and mortar. Valuable insights into how GO can positively influence the durability performance of concrete are summarized, highlighting possibilities for the development of high-performance and durable construction materials using GO. The mechanical properties of GO-induced concrete are equally important when assessing the durability performance of concrete. When enhancing the durability of concrete through the incorporation of GO or other nanomaterials, it is essential to maintain a balance between improving durability and maintaining or even enhancing the mechanical properties of the concrete. The main objective must be driven to achieve a synergistic effect where both durability and mechanical performance are positively influenced. Exposure classifications identify the concentrations of chlorides and sulphates present in soil or groundwater as critical factors in determining the durability of concrete structures. Hence, the chemical resistance of GO-reinforced concrete is also presented in Table 2, which highlights GO’s superior resistance to chloride penetration and sulphate ingress in concrete. 

#### GO as a Catalyst for Hydration

It is well proven that the properties and structure of concrete depend mainly on the hydration reactions of the major cement components present in the cement matrix. Graphene oxide possesses favourable hydration properties such as a high surface area, surface functionalization ability, and excellent dispersibility in aqueous media [94,104,105,106,107]. Comprising mainly hydroxyls and epoxides on its basal planes and carboxyls on the edges, the oxygen-containing functional groups of GO (-OH and -COOH) render the nanomaterial hydrophilic [101,108]. The surface of GO includes rich quantities of these two functional groups, which have two main functions. One of the key functions is to provide active sites to interact with cement, whilst the other is to interact with water molecules by adsorbing them, thus generating water transport channels and reservoirs [95]. C. Lin et al. [109] evaluated the effect of GO on the hydration of cement and discovered that the oxygen-containing functional groups provide adsorption sites for water and cement and that water molecules on GO create water reservoirs and channels for further hydration. Ginigaddara T. et al. [110] studied the effect of using GO synthesized from vein graphite in concrete, assessing the early-age mechanical properties of concrete. The vein graphite used in this study showed 99.5% carbon purity with no impurities and the best crystallinity compared to all the other graphite sources in the world. The experimental data revealed a significant early-strength increment closely associated with the improved degree of hydration. 

The academic literature on GO–cement composites observes that, with increasing concentrations of GO in a cement matrix, the contents of the main hydrates (CSH, CH, Aft) are also increased [108]. The higher zeta potential of GO indicates a stronger interaction between the GO surface and the Ca^2+^ ions [96]. The presence of GO facilitates the mobility of ions, especially Ca^2+^, and, thus, enhances the interaction between the cement surface and the ions. This strong interaction promotes CSH nucleation and growth and, therefore, improves the hydration of cement. The hydration process of cement is critical for delivering the performance-based properties of cement composites. Cement in its anhydrous state mainly consists of Ca_3_SiO_5_ (C_3_S–tricalcium silicate), Ca_2_SiO_4_ (C_2_S–dicalcium silicate), Ca_3_Al_2_O_6_ (C_3_A–tricalcium aluminate), and Ca_4_Aln Fe_2_ nO_7_ (C_4_AF–tetra calcium aluminoferrite) and a small amount of CaSO_4_.2H_2_O (gypsum) and (Na_2_SO_4_, Ka_2_SO_4_) clinker sulphates. When these constituents undergo interactions with water molecules, C_3_S, C_2_S, C_3_A, and C_4_AF undergo a set of complex reactions to form ettringite, calcium hydroxide, and CSH gel, which are the core constituents formed in the initial hydration stage, whilst the contents and crystal shapes of the concrete determine its final strength and durability [38,42,111]. These reactions are summarized in the following equations. In morphology, generally, CH, AFm, and Aft represent needle-like and rod-like shapes in a disorderly manner that highlights the brittle nature of cement.

Ca_3_Al_2_O_6_ + 3CASO_4_.2H_2_O + 26H_2_O Ca_6_Al_2_(SO_4_)_3_(OH)_12_.26H_2_O

2Ca_3_Al_2_O_6_ + Ca_6_Al_2_(SO_4_)_3_(OH)_12_.26H_2_O + 4H_2_O    3Ca_6_Al_2_ (OH)_12_.SO_4_.6H_2_O

2Ca_3_SiO_5_ + 6H_2_O          3CaO.2SiO_2_.4H_2_O + 3Ca(OH)_2_

2Ca_2_SiO_4_ + 4H_2_O          3CaO.2SiO_2_.4H_2_O + 3Ca(OH)_2_

**Figure 5:**
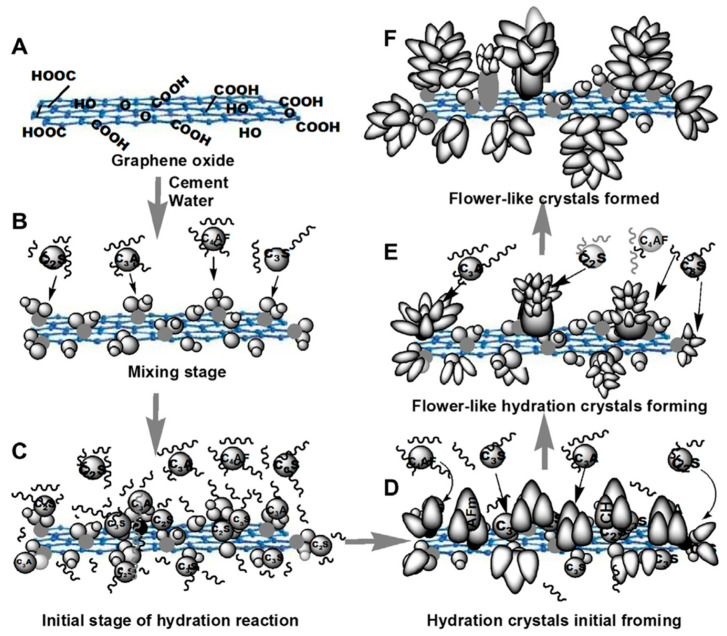
Schematic diagram of the regulation mechanism of GO on cement hydration crystals [34].

Generally, the theory of homogenous nucleation implies that a critical ion concentration must be achieved for the growth of crystal nuclei in a saturated medium [112]. The incorporation of graphene oxide results in heterogenous nucleation, where the critical ion concentration is lowered, thus encouraging nucleation, together with a rapid growth of crystals on the surface of the nanomaterial. Therefore, graphene oxide provides additional nucleation sites for the growth of hydration products, as depicted in Figure 5, which implies that the incorporation of GO accelerates hydration [34].

Therefore, when GO is dispersed in a cementitious matrix, C_3_S, C_2_S, C_3_A, and C_4_AF interact strongly with the functional groups present in GO. These GO-interacted components react effortlessly with the adsorbed water molecules present on the GO, thus giving rise to crystal nucleation sites. The water channels and the reservoirs present in GO accelerate the hydration rate of the cement components. 

## 5. Challenges in the Application of GO-Reinforced Concrete

Despite GO being a promising nano-additive to concrete composites, some underlying challenges hinder the mass-scale adoption of this wonder material. Being discovered in 2004 and winning the Nobel Prize in 2014, graphene is a relatively new material that undergoes rapid technological and research innovations daily. Some of the key challenges associated with GO are the costs associated with a high quality of synthesized GO, safety, dispersibility, stability, and sustainability. It is evident that performance enhancements have a direct correlation with the uniform dispersibility of GO in the cement matrix [95,96,113]. Due to the high surface energy present on a GO lattice, it tends to agglomerate in liquid media, resulting in a reduction in the GO active sites available to participate in nucleation. Furthermore, the high specific surface area of GO sheets encourages the absorption of water molecules to form clusters of GO sheets [93,114]. Moreover, due to their large lateral size, GO sheets tend to have a high water retention capacity, resulting in a reduction in fluidity when exposed to a cementitious matrix [115]. The fluidity of concrete is a critical parameter in commercial-scale applications due to its direct correlation with the workability and pumpability of concrete. A comprehensive toxicology assessment must be undertaken to verify whether GO poses health hazards during handling. However, GO can be easily converted to an aqueous solution due to its hydrophilic nature and can be added to concrete, which hinders the nanomaterial from being transported via air. A lack of specific design standards, field trials, and regulation guidelines is also obstructing the transition of lab-scale GO–concrete trails to commercial-scale applications [99]. With the majority of the studies regarding the durability of GO–concrete being conducted through short-term laboratory experiments, actual long-term field investigations using detailed experimental analyses are important for the development of more realistic predictions.

Cost–benefit assessments are critical when novel engineering materials embrace the commercial market. Despite graphene’s wonderful performance as an additive, its cost must be affordable as well to drive its mass-scale adoption in concrete. However, any relatively new material has significant upfront costs, which can be reduced progressively upon optimizing the manufacturing practices. Nevertheless, the sourcing, processing, functionalization, quality control, supply chain, and logistics of GO must be carefully addressed for this nanomaterial to transform from a technological readiness level to a commercial readiness level. It can also be seen that, despite significant successful lab trials over the past decade, small-, medium-, and large-scale field applications with graphene in concrete are also very scarce. A holistic life-cycle assessment of graphene-based nanomaterials in at least small-scale field trials is of utmost importance to verify its compliance with the relevant building standards.

### Opportunities in GO-Based Concrete Durability Applications

Graphene, undergoing rapid technological progress daily, is giving rise to a wide range of novel applications. As a result, the integration of GO in concrete, aiming to use GO as a successful nano-additive, shows promising future applications. The high sensitivity and conductivity of hybrid GO–concrete opens a wide range of applications in concrete’s self-sensing parameters. Concrete-based sensors are vulnerable to a range of external factors that affect the sensing capabilities of concrete sensors, which can be rectified and safeguarded with the durability enhancement of GO–concrete [30].

Compared to its applications in concrete, the technological advancements in the chemical, biomedical, and electronic-based applications of graphene nanomaterials have matured considerably. The functionalization of graphene has resulted in forceful advancements, opening a wide range of applications of GO. Extracting this knowledge from other engineering disciplines, such as chemical, biomedical, and electronic ones, among others, outlines a great possibility for successful integrations in concrete-based applications. One such example is stabilizing the dispersion of GO in alkaline environments by functionalizing GO with zeolite, nano-SiO_2_ coatings, etc., which shows promising attributes in reducing agglomeration whilst improving durability [87]. Zeolite facilitates the dispersion of nanomaterials in a pore solution by reducing its alkalinity due to the consumption of Ca(OH)_2_. Whilst the durability properties are also improved with the addition of zeolite, the latter leads to a reduction in the mechanical properties, which can be rectified with the addition of GO. GO–zeolite mortars have shown superior performance in resisting chloride and sulphate attacks, having the potential to be used as superior coatings. These factors highlight that GO–zeolite modifications to concrete show promising attributes with respect to confronting dispersion, durability, and mechanical performance [87]. Further studies are required to identify the optimal composition parameters for GO–zeolite–concrete, which will enable it to be applied to concrete structures in harsh environments.

It is reported that GO can improve the antifouling properties (i.e., surface roughness, hydrophobicity, etc.) of materials [116,117]. Marine fouling is also one of the complications that impact the durability of marine concrete structures, generally caused by marine microorganisms. With proper modifications, GO–concrete shows promising attributes in representing itself as an ideal antifoulant carrier [116,118]. Furthermore, added layers of protection on concrete surfaces are also innovative strategies to enhance the durability of concrete structures. GO coatings on concrete surfaces have gained significant interest due to their ability to improve the protection of concrete surfaces, thus enhancing durability. It has been reported that GO-modified epoxy decreases the diffusion coefficient of water by 39.4% and chloride ions by 67.5% [119]. It has also been identified that this enhanced protection is in direct correlation with the amount of GO deposited on the concrete surface [120,121,122]. Hence, detailed investigations are required to identify the optimal coating parameters that provide the finest and most effective protection.

**Table 2:** Durability improvements in graphene oxide-incorporated concrete composites with variable doses and substitutions.

Type	Composition	Improvements	Comments	Ref.
Ground granulated blast furnace slag (GGBFS) concrete with graphene oxide	50 wt.% GGBFS + 50 wt.% PC + 0.1 wt.% GO w/c ratio = 0.40 PC = 212.5 (Kg/m^3^)	42% higher compressive strength (28d) 46% higher compressive strength (90d) RCPT: decrease from 3956 Coulombs (PC) to 1104 Coulombs (GGBFS + GO) (90d)	Increased resistance to chloride penetration Increased corrosion resistance	[123]
Graphene oxide to GBFS–fly ash-based geopolymer concrete	Graphene oxide (0.05 wt.%, 0.15 wt.%, 0.25 wt.%, and 0.35 wt.%) 4% sodium chloride (NaCl) and 0.4 N normality of sodium hydroxide (NaOH) solution	GO-0.25 wt.% +FA65+GBFS35 concrete Compressive strength: 64.27 MPa (56d) Rapid chloride penetrability: 1169 (Coulombs)	Increased resistance to chloride penetration	[124]
Steel fibre-reinforced concrete with graphene oxide	w/c ratio = 0.47 PC = 0.79 (Kg/m^3^)	0.05 wt.% GO + SFRC Compressive strength (28d): 53.3 MPa Cl ion penetration depth: 3.8 mm (56.8% lower than reference specimen) 0.03 wt.% GO + SRFC Cl ion penetration depth: 4.8 mm (53.8% lower than reference specimen)	0.03 wt.% GO shows the best chloride ion resistance out of all the specimens	[67]
Fiber-reinforced composite concrete with graphene oxide	w/c ratio = 0.41 Graphene oxide was added in different proportions (0.03%, 0.06%, 0.09%, and 0.12%) by cement weight	0.12% of GO + 2% SF Compressive strength: 20–56% improvement Decrease percentage in rate of absorption (90d) −45% compared to reference sample 0.03% of GO + 2% SF Decrease percentage in rate of absorption (90d) −17% compared to reference sample	(0.09% GO+ 2% SF) highest strength obtained (90d)	[114]
GO-incorporated concrete for its application in road pavement design	GO at percentages between 0% and 0.08%	0.08%-GO + PCE compressive strength improvement after 7 days −39% compressive strength improvement after 28 days −26% Density of concrete (28 days) 0%-GO+ PCE—2433 kg/m^3^ 0.03%-GO+ PCE—2484 kg/m^3^ 0.08%-GO+ PCE—2493 kg/m^3^	At 0.3% of GO, the volume of abrasion had reduced by 70% compared to the reference sample	[125]
Ultra-high-performance concrete: prepared from recycled sand + graphene oxide (UR)	GO contents of 0.025, 0.05, and 0.075 wt.%	Porosities 0%GO+UR −2.47%, 0.025% GO+UR −2.36%, 0.050% GO+UR −2.19% 0.075% GO+UR −2.25% Most probable pore size 0%GO+UR −11.42 nm 0.025% GO+UR −11.04 nm 0.050% GO+UR −7.38 nm 0.075% GO+ UR −8.23 nm Chloride ion penetration resistance 0%GO+UR −1.16 × 10^−12^ m^2^ s^−1^ 0.025% GO+UR −1.08 × 10^−12^ m^2^ s^−1^ 0.050% GO+UR −1.02 × 10^−12^ m^2^ s^−1^ 0.075% GO+ UR −1.05 × 10^−12^ m^2^ s^−1^	The optimum concentration of GO to be mixed in RS-UHPC was determined to be 0.05 wt.%	[86]
High-strength concrete incorporating wheat straw ash and graphene oxide.	Portland cement + 15% wheat straw ash by cement weight Graphene oxide was added in different proportions (0.060% and 0.080%) by cement weight	0.060 wt.% GO+ 15% WSA Compressive strength improvement: 48.4 to 57.3 MPa (90d) compared to reference sample Water absorption: 14% reduction Sorptivity: 21% reduction Rapid chloride penetrability: 691 Coulombs Acid exposure mass reduction 5.6% (control sample mass reduction: 10.2%)	15% wheat straw ash as a partial substitute for cement and 0.060% inclusion of graphene oxide	[101]
Snail shell-based graphene oxide–cement composite	Effects of substituting snail shell powder for cement at 10, 15, and 20 wt.% GO additions ranged between 0.01, 0.02, 0.03, and 0.04 wt.%	GO-0.04%+SSP-20%: 42.6% increase in compressive strength (28d) GO 0.03%+SSP-20%: water absorption and rapid chloride permeability reduced by 20–40%	Enhanced fluidity of the GO–cement composite Improved resistance to water sorptivity and acidic environmental impacts	[56]
Graphene oxide in ordinary and Portland Pozzolana cement mortars	OPC+ Portland Pozzolana fly ash-based cement (PPC) +(GO) to check the chemical resistance towards 5% sulphuric acid exposure	GO-0.04%+OPC: approx. 24.5% increase in flexural strength (28 days of acid exposure) GO-0.04%+PPC: approx. 1.7% increase in flexural strength (28 days of acid exposure)	GO-reinforced specimens showed better resistance towards acid attacks	[81]
Zeolite-Containing mortars reinforced with graphene oxide	Water to cement in the mixtures (0.485 in all mixtures)	NS and GO: 55MPa (28d) compressive strength ZNS and GO: 53MPa (28d) compressive strength Control sample: 39.4 MPa (28d) compressive strength Compressive strength: approx. 50% increase Expansion rate reduction NS and GO: reduction of 75% ZNS and GO: reduction of 80% in comparison with the control mortar Chloride migration coefficient reduction NS and GO: reduction of 38% ZNS and GO: reduction of 67% in comparison with the control mortar Reduction in porosity NS and GO: reduction of 46% ZNS and GO: reduction of 53.8% in comparison with the control mortar	GO mortars reduced the expansion rate by 56.2% Addition of 15% zeolite in Z mortar reduced the chloride migration coefficient by 40% compared to the control mortar	[87]

## 6. Conclusions 

This review article examines the incorporation of nanomaterials, with a specific emphasis on graphene oxide (GO), within the context of enhancing the durability and performance attributes of concrete. GO promotes cement hydration, provides additional nucleation sites, fills voids, and enhances interfacial bonding, resulting in a refined pore structure with improved durability. The findings presented in this article reveal that the optimal dosage range for graphene oxide (GO) in concrete falls within the parameters of 0.03% to 0.05%. It is noteworthy that the incorporation of GO into the concrete matrix does not exhibit any adverse effects on its overall performance. The incorporation of GO in concrete results in a more compacted microstructure, which demonstrates heightened resistance to the penetration of both chloride and sulphate ions. In some instances, GO–concrete exhibits up to a 50% increase in resistance to chloride ingress when compared to the control sample. Despite the significant improvements shown by GO in enhancing the durability of concrete, the dispersion and retention properties of GO and its stability in highly alkaline cementitious environments require further research and in-depth investigation. The resolution of this issue, if achieved successfully, has the potential to yield notable further improvements in both the mechanical and durability characteristics of concrete through the utilization of GO. This could subsequently allow GO to lead to the consumption of less cement to achieve a similar strength to conventional concrete, consequently advancing the agenda of carbon neutrality and contributing to reductions in emissions. 

In this paper, the influence of GO on improving the durability performance of concrete was comprehensively reviewed. The primary mechanisms involved in the premature deterioration of concrete structures, the transportation mechanisms of harmful agents, the types of chemical attacks, and the cement hydration mechanisms were initially discussed. The durability performance parameters and provisions in AS standards were summarized to provide insights into the design compliance of durable concrete structures. There is a necessity to transform laboratory-based experiments into pilot-scale field trials, which will accelerate the use of nanomaterials in commercial concrete applications. Optimizing the synthesis parameters, surface modifications, and functionalization of GO will enable this wonderful material to push its limits even further, thereby promoting sustainable construction.

## Figures and Tables

**Table 1:** Exposure classifications for concrete in sulphate, acidic, and saline soils extracted from AS 5100.5-2017 [82].

Exposure Conditions			Exposure Classification		
Sulphates (SO_4_)		pH	Chlorides in groundwater ppm	High permeability soils that are in groundwater	Low permeability soils or all soil above groundwater
In Soil ppm	In groundwater ppm				
<1000	<400	>5.5	<2000	B1	A
1000–3000	400–1500	4.5–5.5	2000–8000	B2	B1
3000–20,000	1500–10,000	4–4.5	8000–18,000	C1	B2
>20,000	>10,000	<4	>18,000	C2	C1
